# Excitation of Diverse Classes of Cholecystokinin Interneurons in the Basal Amygdala Facilitates Fear Extinction

**DOI:** 10.1523/ENEURO.0220-19.2019

**Published:** 2019-11-06

**Authors:** Laura Rovira-Esteban, Ozge Gunduz-Cinar, Olena Bukalo, Aaron Limoges, Emma Brockway, Kinga Müller, Lief Fenno, Yoon Seok Kim, Charu Ramakrishnan, Tibor Andrási, Karl Deisseroth, Andrew Holmes, Norbert Hájos

**Affiliations:** 1 Laboratory of Network Neurophysiology, Institute of Experimental Medicine, Hungarian Academy of Sciences, Budapest 1083, Hungary; 2 Laboratory of Behavioral and Genomic Neuroscience, National Institute on Alcohol Abuse and Alcoholism, NIH, Bethesda, MD 20814; 3 János Szentágothai Doctoral School of Neurosciences, Semmelweis University, Budapest 1085, Hungary; 4 Department of Bioengineering, Stanford University, Stanford, CA 94305; 5 Department of Psychiatry and Behavioral Sciences, Stanford University, Stanford, CA 94305; 6 Howard Hughes Medical Institute, Stanford University, Stanford, CA 94305

**Keywords:** basolateral amydala, emotional circuits, inhibitory cells, excitatory cells, mouse

## Abstract

There is growing evidence that interneurons (INs) orchestrate neural activity and plasticity in corticoamygdala circuits to regulate fear behaviors. However, defining the precise role of cholecystokinin-expressing INs (CCK INs) remains elusive due to the technical challenge of parsing this population from CCK-expressing principal neurons (CCK PNs). Here, we used an intersectional genetic strategy in CCK-Cre;Dlx5/6-Flpe double-transgenic mice to study the anatomical, molecular and electrophysiological properties of CCK INs in the basal amygdala (BA) and optogenetically manipulate these cells during fear extinction. Electrophysiological recordings confirmed that this strategy targeted GABAergic cells and that a significant proportion expressed functional cannabinoid CB1 receptors; a defining characteristic of CCK-expressing basket cells. However, immunostaining showed that subsets of the genetically-targeted cells expressed either neuropeptide Y (NPY; 29%) or parvalbumin (PV; 17%), but not somatostatin (SOM) or Ca^2+^/calmodulin-dependent protein kinase II (CaMKII)-α. Further morphological and electrophysiological analyses showed that four IN types could be identified among the EYFP-expressing cells: CCK/cannabinoid receptor type 1 (CB1R)-expressing basket cells, neurogliaform cells, PV+ basket cells, and PV+ axo-axonic cells. At the behavioral level, *in vivo* optogenetic photostimulation of the targeted population during extinction acquisition led to reduced freezing on a light-free extinction retrieval test, indicating extinction memory facilitation; whereas photosilencing was without effect. Conversely, non-selective (i.e., inclusive of INs and PNs) photostimulation or photosilencing of CCK-targeted cells, using CCK-Cre single-transgenic mice, impaired extinction. These data reveal an unexpectedly high degree of phenotypic complexity in a unique population of extinction-modulating BA INs.

## Significance Statement

Distinct types of interneurons (INs) in the basal amygdala (BA) are known to control principal cell activity, allowing complex behaviors. Despite their importance, the role of cholecystokinin (CCK)-expressing inhibitory cells remains unknown. In this work, we could specifically alter the function of CCK-expressing INs in the BA by using an INTRSECT viral strategy. Using a combination of anatomic and electrophysiological methods, we found that CCK^+^ INs in the BA are comprised of cannabinoid receptor type 1 (CB1R)-expressing basket cells, neurogliaform cells, parvalbumin (PV)-expressing basket as well as axo-axonic cells. Importantly, we provided the first direct evidence that CCK-expressing INs in the BA can modulate fear extinction learning. Our data thus show that CCK is expressed in functionally diverse IN populations, positioned to impact amygdala operation.

## Introduction

The basolateral amygdala complex is a neural structure subserving a range of behavioral functions and neural processes, including emotional regulation, and is implicated in the pathophysiology of anxiety and trauma-related disorders (Bukalo et al., 2014; Tovote et al., 2015). The amygdala is comprised of an assortment of cells which differ in their neurochemical identity and efferent and afferent connectivity, but the functional contribution of specific neuronal subpopulations to fear and extinction remains to be fully elucidated (Janak and Tye, 2015). Within the basal nucleus of the amygdala (BA), as in other cortical structures, the activity of principal neurons (PNs) is tightly regulated by local inhibitory GABAergic interneurons (INs) and there is growing evidence that local INs provide a critical regulatory component of the circuits mediating fear and extinction (Ehrlich et al., 2009; McCullough et al., 2016; Krabbe et al., 2018; Lucas and Clem, 2018).

Although INs represent a highly diverse set of cell types, there are evolving efforts to classify them based on their morphologic features, physiologic characteristics and molecular phenotype (DeFelipe et al., 2013). One commonly adopted method segregates IN subpopulations based on neurochemical content, including expression of Ca^2+^-binding proteins [e.g., parvalbumin (PV); McDonald and Betette, 2001; McDonald and Mascagni, 2001b] and neuropeptides such as somatostatin (SOM), neuropeptide Y (NPY), and cholecystokinin (CCK; Mascagni and McDonald, 2003; Kepecs and Fishell, 2014). In turn, there is emerging evidence for critical contributions of some of these subpopulations to fear behaviors. For example, an elegant series of studies has shown that PV-containing and SOM-containing BA INs act in concert to gate the responses of PNs to conditioned stimulus (CS) and unconditioned stimulus (US) during fear memory acquisition (Wolff et al., 2014). However, the possible roles of other subpopulations of BA INs, including NPY-expressing (Mańko et al., 2012) and CCK-expressing (Rovira-Esteban et al., 2017), in regulating fear and extinction remain unclear.

Adding to the complexity of studying CCK INs, within the BA these cells appear to fall into subclasses that are differentially positioned within the broader extinction circuitry. In rodent BA, CCK INs with large soma (CCK-L) express either vesicular glutamate transporter type 3 (VGluT3) or Ca^2+^ binding protein, calbindin (Calb) and are distingushed from NPY-expressing CCK INs with small soma (CCK-S) (Mascagni and McDonald, 2003; Omiya et al., 2015; Vogel et al., 2016; Rovira-Esteban et al., 2017; Veres et al., 2017). BA CCK-L INs make functionally-potent perisomatic connections onto BA PNs (Vereczki et al., 2016; Andrási et al., 2017; Barsy et al., 2017; Veres et al., 2017) that in turn project to the dmPFC and vmPFC (Vogel et al., 2016). Interestingly, extinction recruits CCK-L INs targeting the extinction-constraining BA→dmPFC pathway (Trouche et al., 2013; Senn et al., 2014; Vogel et al., 2016). Moreover, CCK-L express the cannabinoid receptor type 1 (CB1R) on their axonal varicosities (Marsicano and Lutz, 1999; Katona et al., 2001; McDonald and Mascagni, 2001a; Mascagni and McDonald, 2003; Yoshida et al., 2006; Bowers and Ressler, 2015). Of note here, previous studies have shown that cannabinoid signaling at BA CB1Rs promotes fear extinction (Marsicano et al., 2002; Chhatwal et al., 2005; Gunduz-Cinar et al., 2013), an effect that is known to depend at least to some extent on receptors expressed at GABAergic axon terminals (Ruehle et al., 2013).

Taken together, the current literature support the hypothesis that CCK INs affect extinction by dynamically adjusting the balance of inhibitory control over opposing BA output-pathways (Vogel et al., 2016). In this context, the goal of the current study was to further characterize the electrophysiological, morphological and molecular properties of CCK INs in the BA and assess their contribution to fear extinction. To this end, we employed an intersectional approach (Fenno et al., 2014) to genetically access and manipulate BA CCK INs (Taniguchi et al., 2011; Senn et al., 2014; Whissell et al., 2019).

## Materials and Methods

### Subjects

C57BL/6J (JAX stock #000664), CCK-IRES-Cre (*Cck^tm1.1(cre)Zjh^*/J, JAX stock #012706) and Dlx5/6-Flpe (Tg(mI56i-flpe)39Fsh/J, JAX stock #010815) mice were obtained from The Jackson Laboratory. Homozygous Cck-Cre and homozygous Dlx5/6-Flpe mice were bred to produce Cck^Cre^;Dlx5/6^Flp^ (CCK IN) mice. Males and females were used for electrophysiological recordings and male mice were used for behavioral testing. Mice were housed in same-sex groupings (two to four per cage); mice with chronic fiber implantations for *in vivo* optogenetics were single-housed after surgery to prevent cage-mates damaging the cranial implants. Housing was in a temperature- and humidity-controlled vivarium under a 12/12 h light/dark cycle (lights on 6 A.M.). Experiments were conducted during the light phase.

All experimental procedures were performed in accordance with the Institutional Ethical Codex, Hungarian Act of Animal Care and Experimentation (1998. XXVIII. section 243/1998, renewed in 40/2013), the European Union guidelines (directive 2010/63/EU), the National Institute of Health (NIH) Guide for the Care and Use of Laboratory Animals and approved by the Institutional Animal Care and Use Committee of the Institute of Experimental Medicine and the local National Institute on Alcohol Abuse and Alcoholism (NIAAA) and Vanderbilt Animal Care and Use Committees.

### Stereotaxic surgery

Mice were placed in a stereotaxic frame (David Kopf Instruments) to bilaterally inject viral constructs into the BA (coordinates: anterior-posterior –1.4 to 1.5 mm, medial-lateral ±3.22 to 3.3 mm, dorsal-ventral –4.4 to 4.85 mm to bregma). Virus was injected in a volume of 0.2 µl per hemisphere at a rate of 3 nl/s (for *ex vivo* optogenetics) or in a volume of 0.4–0.5 µl per hemisphere over 10 min (for *in vivo* optogenetics), according to each laboratory’s local practices and pilot work. Injections were done using a 1-µl syringe (Neuros model 7001 KH, Hamilton Robotics) connected to a UMP3 UltraMicroPump and SYS-Micro4 Controller or Nanoliter NL2010MC4 injector (World Precision Instruments, LLC). The syringe was left in place for an additional 5 min to ensure constructs diffused into the tissue. For *in vivo* optogenetics, during the same surgery as viral injections, ferrules and 200-µm diameter fiber optics (numerical aperture, 0.37) were bilaterally inserted into the BA and affixed to the skull with dental cement. The ferrule-fiber assembly was constructed according to previously published methods (Bukalo et al., 2015; Bergstrom et al., 2018; Radke et al., 2019).

### Viral constructs

Adenoassociated virus (AAV)-based constructs engineered to transfect Cre+ cells with channelrhodopsin-2 (ChR2; AAV5-EF1a-DIO–hChR2(H134R)-EYFP), archaerhodopsin (eArch3.0; AAV5-EF1a-DIO-eArch3.0-EYFP), or control vector (AAV5-EF1a-DIO-EYFP) were obtained from the University of North Carolina Vector Core. The AAV-based INTRSECT (INTronic Recombinase Sites Enabling Combinatorial Targeting)-related constructs engineered to transfect Cre+/Flp+ cells with ChR2 (AAVdj-hSyn-Con/Fon-hChR2(H134R)-EYFP-WPRE in *ex vivo* experiments, and pAAV-nEF1-Con/Fon-hChR2(H134R)-EYFP-WPRE in behavior experiments, hereafter referred to as INTRSECT-ChR2), Arch3.3 (AAVdj-hSyn-Con/Fon-Arch3.3-EYFP, hereafter referred to as INTRSECT-Arch) or a control virus nEF-Con/Fon-eYFP-WPRE were obtained from the University of North Carolina Vector Core or directly from the Deisseroth laboratory. The virus titers were 3–6 × 10e12 vg/ml.

### Fluorescence *in situ* hybridization

At least five weeks after delivery of AAVdj-hSyn-Con/Fon-hChR2(H134R)-EYFP-WPRE, CCK IN mice were killed by cervical dislocation, then brains were immediately removed and frozen in 2-methyl butane on dry ice and stored at –80°C. Coronal sections, 16-μm-thick, were cut using a cryostat (model HM500 OM, Microm International GmbH) and mounted directly onto Super Frost Plus slides (Fisher Scientific) and maintained at –20°C before transfer to a staining jar containing 4°C 10% buffered formalin solution. After a 20-min fixation, slides were rinsed twice in PBS for 1 min each, then dehydrated in an ethanol dilution series (50%, 70%, and 100% ×2) and stored at –20°C overnight in 100% ethanol.

The following day, the sections were processed using an RNAscope Fluorescent Multiplex Assay kit (Advanced Cell Diagnostics USA; Gunduz-Cinar et al., 2019) according to the manufacturer’s instructions. Slides were first air dried for 10 min, then a hydrophobic barrier drawn around each section with an ImmEdge barrier pen (Vector Laboratories) to limit the spread of solutions. The sections were treated with Pretreat-4 protease solution for 20 min at room temperature and slides washed twice with distilled water. In one set of samples, target probes for Mm-*Cck* (gene ID 12424, catalog #402271) and Mm-*Gad1* (glutamate decarboxylase 1, GAD-67; gene ID: 14415, catalog #400951-C2) were spread evenly with a pipette tip on the sections, and the slides were incubated at 40°C for 2 h in a hybridization oven (model HybEZ, Advanced Cell Diagnostics). A second set of samples were treated with Mm-*Cck* and Mm-*Slc17a7* (VGluT1; gene ID 72961, catalog #416631-C2). These probes and those described below were obtained from Advanced Cell Diagnostics.

Sections were next treated with amplifier and fluorescent probes to separately label each gene (AMP1 at 40°C for 30 min; AMP2 at 40°C for 15 min, AMP3 at 40°C for 30 min, and AMP4 AltB at 40°C for 15 min). Slides were washed twice with 1× wash buffer according to the manufacturer’s guidelines between incubation steps. Finally, the sections were incubated for 20 s with DAPI at room temperature, and then coverslipped with Vectashield HardSet fluorescent mounting medium (Vector Laboratories). For each brain, separate sections were processed as either (1) a negative control to confirm the absence of background labeling on each channel using a bacterial mRNA (DapB of Bacillus subtilis strain; 3-plex Negative Control Probe, catalog #320871), or (2) a positive control to confirm the ability to detect the presence of labeling on each channel, using three housekeeping genes [RNA polymerase II subunit A (*Polr2a*), peptidylprolyl isomerase B (*Ppib*), ubiquitin C (*Ubc*); 3-plex Positive Control Probe, catalog #320881]. Images of the BA were obtained on 405-, 488-, 550-nm fluorescent channels using a confocal microscope (model LSM 700, Carl Zeiss Microscopy LLC) under a Plan-Apochromat 20×/0.8 M27 objective.

### Immunohistochemistry

#### Immunostaining for GABAergic and glutamatergic markers

At least five weeks after delivery of AAVdj-hSyn-Con/Fon-hChR2(H134R)-EYFP-WPRE, mice were terminally overdosed with ketamine/xylazine and transcardially perfused first with saline followed by ice cold 4% paraformaldehyde (PFA) in phosphate buffer, and after removing the brain from the skull an overnight postfixation took place at 4°C. Coronal sections (50-μm-thick) were cut using a vibratome (model VT1000S, Leica Biosystems) in 0.1 M phosphate buffer and incubated in 0.8% sodium borohydride for 30 min. The sections were rinsed in PBS and blocked for 2 h in a solution containing 0.2% Triton X-100, 10% normal goat serum, and 2% bovine serum albumin. Sections were then rinsed and incubated overnight at 4°C with chicken anti-green fluorescent protein (GFP; 1:1000, catalog #ab13970, Abcam)/mouse anti-CCK (1:250, catalog #CCK8-MO-167-1, Frontier Institute), GFP/mouse anti-Ca^2+^/calmodulin-dependent protein kinase II (CaMKII; 1:500, catalog #10011437, Cayman Chemical), or GFP/mouse anti-Gad67 (1:500, catalog #MAB5406, MilliporeSigma, or catalog #ab26116, Abcam), GFP/rabbit anti-Gad67 (1:500, catalog #Af260, Frontier Institute) in PBS containing 0.2% Triton X-100, 1% normal goat serum, 0.1% sodium azide.

The next day, sections were re-rinsed and incubated for 2 h at room temperature in either (for the GFP/CCK combination) Alexa Fluor 488 (goat anti-chicken, 1:2000, catalog #A11039, Life Technologies)/Alexa Fluor 555 (goat anti-rabbit, 1:2000, catalog #A21428, Life Technologies), or (for the GFP/Gad67 and GFP/CaMKII combinations) Alexa Fluor 488 (goat anti-chicken, as above)/Alexa Fluor 555 (goat anti-mouse, 1:2000, catalog #A21422, Life Technologies). The sections were then re-rinsed, mounted, and coverslipped with Vectashield HardSet mounting media (Vector Laboratories). Images of the BA were obtained on 405-, 488-, 550-nm fluorescent channels using a confocal microscope (model LSM 700, Carl Zeiss Microscopy) under a Plan-Apochromat 20×/0.8 M27 and Plan-Apochromat 63×/1.40 Oil DIC objectives.

#### Immunostaining for IN subtype markers

At least five weeks after delivery of AAVdj-hSyn-Con/Fon-hChR2(H134R)-EYFP-WPRE, mice were terminally overdosed with ketamine/xylazine and transcardially perfused first with saline followed by ice cold 4% PFA in phosphate buffer. After removing the brain from the skull postfixation took place at 4°C for 3 h. Coronal sections (50-μm-thick) were cut using a vibratome (model VT100S, Leica Biosystems).

To reveal the immunoreactivity for different markers, sections containing the BA were further processed for immunostaining with the following antibody mixtures: rabbit anti-CaMKII (1:1000, catalog #ab52476, Abcam) and guinea pig anti-PV (1:10,000, catalog #195004, Synaptic Systems), rabbit anti-NPY (1:500 courtesy of Prof. Günther Sperk) and guinea pig anti-nNOS (1:1000, catalog #nNOS-GP-Af740, Frontier Institute), rabbit anti-NPY (1:500) and guinea pig anti-PV (1:10,000), or rabbit anti-PV (1:5000, catalog #PV25, Swant Antibodies, Marly, Switzerland) and guinea pig anti-Calb (1:3000, catalog #14004, Synaptic Systems).

To visualize these antibodies, Cy3-conjugated donkey anti-rabbit antibody (1:500, catalog #711-166-152, Invitrogen) and Cy5-conjugated donkey anti-guinea pig (1:500, catalog #706-175-148, The Jackson Laboratory) were used. In addition, we incubated different sections in rat anti-SOM (1:500, catalog #MAB354, MilliporeSigma) or in rabbit anti-CB1R, which stains only the CB1R-expressing GABAergic axon terminals (1:1000, catalog #10006590, Cayman Chemicals). The localization of these antigens was visualized with Cy3-conjugated donkey anti-rat (1:500, catalog #712-165-153, Jackson ImmunoResearch) or DyL405-conjugated goat anti-rabbit (1:500, catalog #111-475-003, Jackson ImmunoResearch). A confocal microscope (model C2, Nikon Instruments Europe BV) was used to obtain images of soma (under a Plan-Apochromat 20× objective (N.A. 0.75, *z* step size: 1 µm, *xy*: 0.62 µm/pixel) and axon terminals (under a Plan-Apochromat VC 60× objective (N.A. 1.4, *z* step size: 0.5 µm, *xy*: 0.21 µm/pixel). Those neurons that showed EYFP immunoreactivity in both the cytoplasm and cell membrane were considered EYFP+ and could be clearly distinguished from those EYFP- neurons that soma and proximal dendrites were surrounded by axonal varicosities expressing EYFP.

### *Ex vivo* optogenetics

#### Electrophysiological slice recordings

Ten to 12 weeks after injection of the INTRSECT-ChR2 virus, mice were deeply anesthetized with isoflurane, the brain was quickly removed and placed into ice-cold solution containing: 252 mM sucrose, 2.5 mM KCl, 26 mM NaHCO_3_, 0.5 mM CaCl_2_, 5 mM MgCl_2_, 1.25 mM NaH_2_PO_4_, 10 mM glucose, bubbled with 95% O_2_/5% CO_2_ (carbogen gas). Horizontal 200-µm thick brain sections containing the BA were prepared with a vibratome (model VT1200S, Leica Biosystems) and kept in an interface-type holding chamber containing ACSF at 36°C that gradually cooled down to room temperature. ACSF contained the following: 126 mM NaCl, 2.5 mM KCl, 1.25 mM NaH_2_PO_4_, 2 mM MgCl_2_, 2 mM CaCl_2_, 26 mM NaHCO_3_, and 10 mM glucose, bubbled with carbogen gas. After at least a 60-min-long incubation, slices were transferred to a submerged-type recording chamber and perfused with 32–34°C ACSF with a flow rate of 2–2.5 ml/min.

Recordings were performed under visual guidance using differential interference contrast microscopy (via a model FN-1 Nikon upright microscope) using 40× water dipping objective. EYFP expression was visualized with the aid of a mercury arc lamp and a CCD camera (Andor Technology). Neurons that did not express EYFP were recorded where EYFP+ fibers were densest. Patch pipettes (5–7 MΩ) for whole-cell recordings were pulled from borosilicate capillaries with inner filament (thin walled, OD 1.5) using a P1000 pipette puller (Sutter Instrument). In whole-cell recordings the patch pipette contained a K-gluconate based intra-pipette solution containing the following: 115 mM K-gluconate, 4 mM NaCl, 2 mM Mg-ATP, 20 mM HEPES, 0.1 mM EGTA, 0.3 mM GTP (sodium salt), and 10 mM phosphocreatine adjusted to pH 7.3 using KOH, with an osmolarity of 290 mOsm/l. The pipette also contained 0.2% biocytin. Recordings were performed with a Multiclamp 700B amplifier (Molecular Devices), low-pass filtered at 3 kHz, digitized at 10 kHz, recorded with Clampex 10.4 (Molecular Devices), and were analyzed with Clampfit 10.4 (Molecular Devices) and OriginPro 2015 (OriginLab Corp).

Whole-field blue light (447 nm) laser illumination (Roithner Laser Technik, Vienna, Austria) was applied for 100 ms using a Digital Mirror Device based pattern illuminator (Mightex Polygon 400, Mightex Systems) to activate even those neurons expressing ChR2 that membrane time constants are rather slow. The recorded neurons were clamped at a holding potential of –50 mV. Series resistance was in the range of 15–25 MΩ. For peak and area analysis, five consecutive traces were averaged. Drug effects were evaluated after a 10-min wash-in of bath-applied gabazine (5 µM), CGP 5699A (1 µM), and CP 55,940 (2 µM), or after 20-min wash-in of bath-applied AM251 (2 µM). The peak amplitude for the fast and slow components was determined at distinct time points (*n* = 23). The area of these two components was calculated only for those events in which the fast component was blocked by gabazine (*n* = 10): the remaining slow component was subtracted from the original trace resulting in the area for the fast component. For firing pattern analyses, EYFP+ neurons were recorded in current clamp mode at a holding potential of –65 mV. Voltage responses were tested to a series of hyperpolarizing and depolarizing square current pulses of 800-ms duration and amplitudes between –100 and 100 pA at 10-pA step intervals, then up to 300 pA at 50-pA step intervals and finally up to 600 pA at 100-pA step intervals.

### Postrecording IN subtype identification

Biocytin content was visualized using Cy3-conjugated streptavidin (1:10,000, catalog #S6402, Sigma-Aldrich) and confocal images of the filled cells were obtained using a confocal microscope (Nikon model C2) under a Plan-Apochromat VC 20× objective (N.A. 0.75, *z* step size: 1 µm, *xy*: 0.40 µm/pixel). Cells were immunostained with antibodies selected on the bases of a combination of their firing characteristics and the features of their dendritic and axonal arbors. Putative CCK^+^ basket cells were immunostained with goat anti-CB1R antibody (1:1000, catalog #CB1-Go-Af450, Frontier Institute) and visualized using DyL405-conjugated donkey anti-goat antibody (1:500, catalog #705-475-003, Jackson ImmunoResearch). Only those cells (five of seven) expressing CB1R in their axonal terminals were categorized as CCK^+^ basket cells.

Putative fast spiking PV+ cells were immunostained with rabbit anti-PV (1:5000, catalog #PV25, Swant) visualized with A647-conjugated donkey anti-rabbit (1:500, catalog #711-605-152, Jackson ImmunoResearch), and chicken anti-Calb (1:5000, catalog #214006, Synaptic Systems) visualized with DyL405-conjugated donkey anti-chicken (1:500, catalog #703-475-15, Jackson ImmunoResearch). Confocal images (see below) were taken to assess Calb and PV co-expression at axonal terminals. Those cells (three of nine) co-expressing Calb and PV at their axonal boutons were categorized as PV+ basket cells, whereas those cells (six of nine) which expressed PV, but not Calb, at their axon terminals were considered axo-axonic cells.

To further confirm the latter classification, *in vitro* slices containing both PV+ basket cells and axo-axonic cells were re-sectioned into 30 µm-thick sections, pepsin digested as described previously (Veres et al., 2014) and immunostained using a mouse anti-ankyrin G antibody (1:100, catalog #75-146, NeuroMab) visualized with an Alexa Fluor 488-conjugated donkey anti-mouse antibody (1:500, catalog #A21202, Millipore). Those cells which showed cartridges of axonal terminals in close apposition to ankyrin G+ profiles were confirmed as axo-axonic cells. Because the cells selected for recording and subsequent biocytin filling based on being EYFP+, there was a low level of EYFP fluorescence in their axonal terminals. In these images, the red channel containing the signal of the Cy3-conjugated streptavidin used to visualize the biocytin was subtracted from the green channel, and the axonal terminal apposition to ankyrin G+ profiles was evaluated in these modified images merged with the original image taken in red channel. All images were obtained using a confocal microscope (Nikon model C2) under a Plan Apo VC 60× objective (N.A. 1.4, *z* step size: 0.15–0.2 µm, *xy*: 0.08–0.10 µm/pixel).

### *In vivo* optogenetics

#### *In vivo* photostimulation and photosilencing

For photostimulation, blue light (λ = 473 nm) was bilaterally shone on the BA at 20 Hz, in 5-ms pulses, consistent with commonly used ChR2-excitation parameters and mimicking the activity of highly active neurons. For photosilencing, green light (λ = 532 nm) was bilaterally shone on the BA continuously, which is also in line with commonly used Arch-excitation parameters. For INTRSECT experiments in CCK IN mice, laser power was set to 7 mW both for blue and green laser, measured at the tip of optic fiber. For the ChR2-photostimulation experiment in CCK-Cre mice, the laser power was reduced to 3 mW after pilot data showed 7-mW produced indications of seizure activity. Laser power was calibrated before each experiment by measuring the power at the tip of the patch cord with a PM100D optical power meter with an S120C sensor (Thorlabs) and multiplying that power by the transmittance of the ferrule connection on each optic fiber.

### Behavioral testing

Fear conditioning and extinction testing was conducted (Whittle et al., 2010) at least four weeks after virus delivery. Before testing, each mouse was handled for 2 min/d for 6 d and habituated to the connected optic-fiber cables in the home cage for 40 min/d for 3 d.

#### Fear conditioning

Fear conditioning was conducted in context A: a 30 × 25 × 25 cm operant chamber (Med Associates, Inc.) with metal walls and a metal rod floor. To provide an additional olfactory cue, the chamber was cleaned between subjects with a 79.5% water: 19.5% ethanol: 1% vanilla extract solution. Beginning after a 120- to 180-s stimulus-free period, there were 3× pairings (60- to 90-s interpairing interval) between a 30-s, 80-dB white noise cue (CS) and a 2-s 0.6-mA scrambled co-terminating footshock (US) followed by a 120-s stimulus-free period. The Med Associates Video Freeze Monitor System controlled CS and US presentation.

#### Fear extinction training

Fear extinction training occurred the following day in context B: a 27 × 27 × 14 cm operant chamber with transparent walls and a floor covered with wood chips, cleaned between subjects with a 99% water:1% acetic acid solution and housed in a different room from training. After a 180-s stimulus-free baseline, there were 50 × 30-s CS presentations (5-s inter-CS interval). During each CS, light (blue for photostimulation, green for photosilencing) was shone on the BA.

#### Extinction retrieval

Extinction retrieval was tested the day after extinction training in context B, via 5 × 30-s no-light CS presentations (5-s inter-CS interval) beginning after a 180-s stimulus-free baseline. Mice were connected to the fiber cables during the retrieval test, to control for the influence of a potential extinction-context cue.

Freezing, the absence of any visible movement except respiration, was scored from video every 5 s throughout testing by an experienced observer blind to genotype. The mean number of freezing observations per baseline period and 5× CS block was converted to a percentage [(number of freezing observations/total number of observations per period) × 100] for analysis. Mice with freezing scores on any CS-block that were >3 SDs from the group mean were excluded from the analysis.

#### Novel open field test

Mice were tested in the novel open field test for exploratory locomotion and anxiety-like behaviors (Fitzgerald et al., 2010) 7 d after the completion of fear and extinction testing. The apparatus was a square arena (39 × 39 × 35 cm) with opaque white Plexiglas walls and floor (∼95 lux). Mice were connected to the fiber optic cables and placed in the center for a 10-min test session, during which light (blue for photostimulation, green for photosilencing, as above) was shone on the BA for 2 × 2-min periods, interspersed with 2-min light-off periods (i.e., off-on-off-on-off). Total distance traveled, average movement speed and time spent in a 25-cm^2^ center zone was measured by the EthovisionXT videotracking system (Noldus Information Technology).

### Postbehavior confirmation of fiber placement and virus expression

At the completion of behavioral testing, mice were terminally overdosed with ketamine/xylazine and transcardially perfused with PBS, then 4% PFA. After overnight suspension in 4% PFA, followed by 0.1 M phosphate buffer for 1–2 d at 4°C, 50-μm coronal sections were cut with a vibratome (Leica Biosystems). Sections were mounted and coverslipped with Vectashield HardSet mounting medium with 4′,6-diamidino-2-phenylindole (Vector Laboratories) and imaged with a fluorescent (model BX41, Olympus America, Inc.) or confocal microscope (model LSM 700, Carl Zeiss Microscopy) under a Plan-Apochromat 20×/0.8 M27 objective.

### Statistical analysis

Group effects in electrophysiological experiments were analyzed using paired *t* test. For scatterplots, each symbol represents the mean average of five consecutive events. Group effects in behavioral experiments were analyzed using Student’s *t* test or ANOVA, depending on the number of groups, followed by Fisher’s LSD *post hoc* tests paired Student’s *t* test. The threshold for statistical significance was set at *p* < 0.05. Values given in the Results represent the mean ± SEM.

## Results

### Intersectional strategy for neuronal targeting

**Figure 1.**
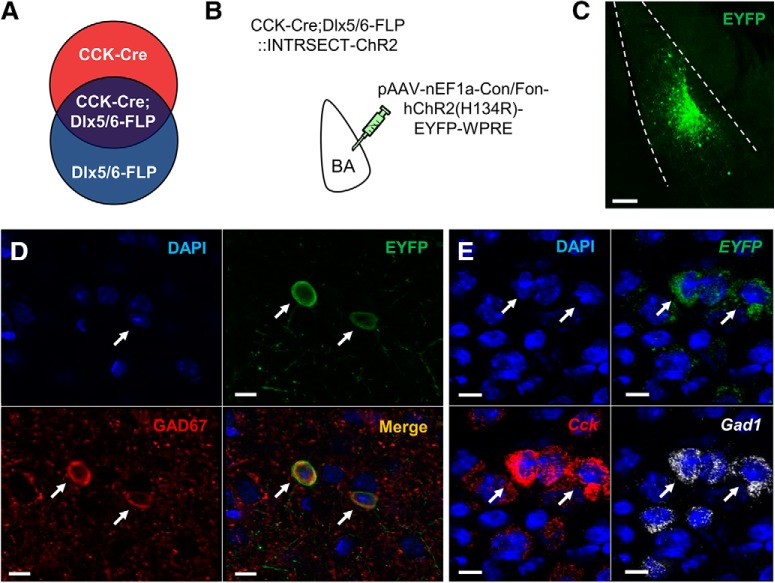
Intersectional strategy for targeting BA INs. ***A***, Schematic of the intersectional strategy used to target BA INs in CCK-Cre;Dlx5/6-Flp double-transgenic mice with (***B***) INTRSECT pAAV-nEF1a-Con/Fon-hChR2(H134R)-EYFP-WPRE virus. ***C***, Representative example of EYFP expression after virus transfection (scale bar = 100 µm). ***D***, Virus-transfected (EYFP-expressing) cells immunopositive for GAD67 (white arrows, scale bar = 10 µm). ***E***, Virus-transfected (EYFP-expressing) cells labeled with *Cck* and *Gad1* mRNA; arrows denote two example neurons positive for *EYFP*, *Cck*, and *Gad1* (white arrows, scale bar = 10 µm).

To gain selective genetic access to CCK INs, a double-transgenic mouse line was generated by intercrossing a CCK-Cre driver line with a Dlx5/6-Flpe driver line, which expresses Flp recombinase in most cortical GABAergic neurons (Miyoshi and Fishell, 2011; Taniguchi et al., 2011; Vogel et al., 2016; Fig. 1*A*). We then used an intersectional optogenetic viral strategy to visualize and control the targeted cells, involving the bilateral injection of CCK IN double-transgenic mice with INTRSECT viruses transfecting cells with EYFP-tagged ChR2 in a manner conditional on the presence of both Cre and Flp recombinases (Fenno et al., 2014; Fig. 1*B*,*C*). Initial, non-quantitative immunocytochemical staining for the GABAergic neuronal marker, GAD67, indicated co-localization with EYFP cells in the BA (Fig. 1*D*). Similarly, fluorescence *in situ* hybridization showed that EYFP expression appeared concordant with the labeling of BA cells that were also positive for *Cck* and *Gad1* mRNA probes (Fig. 1*E*).

### Targeted neurons are GABAergic

**Figure 2.**
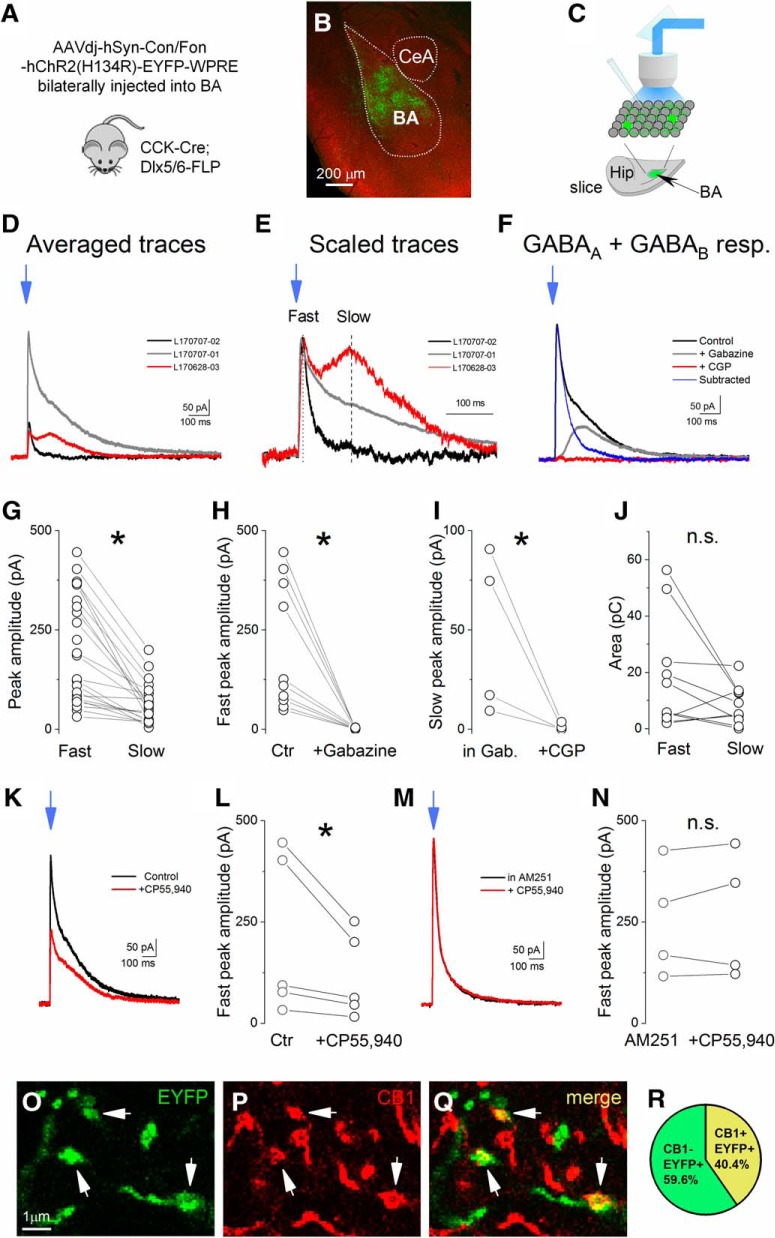
Photostimulation of GABAergic cells in the BA. ***A***, Schematic drawing showing the intersectional viral strategy used to target CCK^+^ INs in CCK-Cre;Dlx5/6-FLP double-transgenic mice. ***B***, Example of INTRSECT AAVdj-hSyn-Con/Fon-hChR2(H134R)-EYFP-WPRE (INTRSECT-ChR2) expression in the BA (CeA = central amygdala). ***C***, Schematic drawing represents a horizontal slice with viral expression shown in green (Hip = hippocampus). Whole-cell patch clamp recordings were performed in non-green cells, likely in PNs in slices prepared from double-transgenic mice injected with AAV containing INTRSECT-ChR2. ***D***, ***E***, Averaged traces of five consecutive PSCs obtained in three different neurons evoked by light illumination (blue arrow). High variability both in peak amplitude (***D***) and decaying phase (***D***, ***E***) is typical for events evoked in different neurons. The traces in ***E*** are peak scaled. Dashed lines show where the peak amplitude for fast and slow components of evoked currents was measured. ***F***, Traces from an experiment measuring the antagonist-sensitivity of light-evoked responses. Gabazine (5 µM) wash-in eliminated the fast GABA_A_-mediated component, while CGP 5699A (1 µM) blocked the remaining slow GABA_B_-mediated component. Importantly, no inward, i.e., EPSC, could be observed in the presence of the GABA receptor antagonists, indicating that the applied intersectional strategy allowed us to excite selective GABAergic cells. ***G***, Peak amplitude of the fast components in evoked responses measured in the same neurons was significantly larger than the peak amplitude of the slow components. ***H***, The fast components were blocked by bath application of gabazine (*paired *t* test). ***I***, The slow components were eliminated by CGP 5699A wash-in. ***J***, The area, i.e., the charge of the fast and slow components evoked in the same neurons, was not different. GABA_A_ receptor-mediated fast responses were isolated by subtracting the responses recorded in the presence of gabazine from the control traces and their area was measured. Example traces (subtracted) are shown in ***F***. The area of the GABA_B_ receptor-mediated slow components were determined on the traces recorded in the presence of gabazine. ***K***, Averaged traces taken from an example experiment indicate that the light-evoked PSCs are smaller on wash-in of a CB1R agonist, CP 55,940 (2 µM). ***L***, In all experiments tested, bath application of CP 55,940 significantly reduced the peak amplitude of the fast component. ***M***, Averaged traces taken from an experiment showing that, in the presence of the CB1R antagonist, AM251 (2 µM), bath application of CP 55,940 (2 µM) did not cause a reduction in the peak amplitude. ***N***, Preincubation of the slices in AM251 prevented the CP 55,940-induced reduction of the peak amplitude of light-evoked postsynaptic responses. ***O–Q***, A portion of EYFP-expressing axon terminals is immunoreactive for CB1 (arrows). ***R***, Approximately 40% of EYFP-expressing axonal varicosities were immunopositive for CB1R (156 EYFP+ varicosities were tested in two mice); *p < 0.05 fast versus slow, +gabazine versus control (Ctr), +CGP versus in gabazine (Gab), +CP versus Ctr. n.s., non-significant.

To further assess the selectivity of the intersectional targeting approach employed, we performed *ex vivo* electrophysiological recordings in CCK IN mice transfected with the INTRSECT-ChR2 virus (Fig. 2*A–C*). Whole-cell recordings from EYFP-negative, likely PNs (*n* = 23), which were postsynaptic to EYFP-expressing neurons in BA-containing brain slices, revealed that blue light illumination evoked outward currents, but with substantial variance both in the peak amplitude and decaying phase (Fig. 2*D*,*G*). Further inspection of these responses indicated that in some cases, the light-evoked currents clearly had fast and slow components, recognized by distinct peaks (Fig. 2*E*). The two outward components had significantly different peak amplitude (fast component: 201.6 ± 27.8 pA, slow component: 63.6 ± 10.7 pA, *n* = 23, *t*_(43)_ = 4.55, *p* < 0.001; Fig. 2*G*), but they carried similar charge (fast component: 18.4 ± 6.2 pC, slow component: 8.4 ± 2.2 pC, *n* = 10, *p* = 0.1; Fig. 2*J*).

We then performed pharmacological manipulations showing that the fast component was blocked by the GABA_A_ receptor antagonist, gabazine (peak amplitude in control: 202.3 ± 50.3 pA; in gabazine: 2.0 ± 0.5 pA, *n* = 10, *t*_(9)_ = 3.99, *p* = 0.003; Fig. 2*F*,*H*). Conversely, the remaining slow outward current was eliminated by the GABA_B_ receptor antagonist, CGP 5699A (peak amplitude in gabazine: 47.9 ± 20.4 pA, in CGP 5699A: 1.2 ± 0.8 pA, *n* = 4, *t*_(3)_ = 2.38, *p* = 0.04; Fig. 2*F*,*I*). No inward (i.e., excitatory synaptic current) was evident in any recordings, even in the presence of both GABA receptor antagonists; indicating that photostimulation exclusively evoked inhibitory, GABA receptor-mediated synaptic currents in BA neurons, consistent with the selective targeting of INs, and not PNs, in this region.

### Targeted neurons express functional CB1R

Based on earlier reports that CCK-containing basket cells in the BA express CB1R, we next asked whether the targeted BA INs were CB1R-positive (Katona et al., 2001; McDonald and Mascagni, 2001a; Vereczki et al., 2016; Veres et al., 2017). To test the responsivity of the targeted cells to CB1R activation, we generated light-evoked postsynaptic currents (PSCs) in EYFP-negative cells followed by bath application of the CB1R agonist, CP 55,940. The application of the agonist reduced the peak amplitude of light-evoked events by 50% (control: 209.8 ± 88.4 pA, in CP: 112.6 ± 47.6 pA, peak ratio: CP/ctr: 51.3 ± 5.4%, *n* = 5, *t*_(4)_ = 2.35, *p* = 0.039; Fig. 2*K*,*L*). We verified that the CP 55,940-induced reduction was CB1R mediated by abolishing the effect via preincubation with the CB1R antagonist, AM251 (peak amplitude in AM251: 261.8 ± 70.2 pA, in AM251 + CP 55,940: 259.5 ± 78.4 pA, peak ratio: AM251 + CP 55,940/AM251: 96.7 ± 8.9%, *n* = 4, *p* = 0.89; Fig. 2*M*,*N*). These data demonstrate that a significant component of synaptic currents in BA neurons evoked by light illumination in slices stems from signaling through the CB1R. Consistent with these findings, immunostaining BA sections containing transfected processes revealed that ∼40% of EYFP-expressing axonal varicosities were positive for CB1R (Fig. 2*O–R*). These results indicate that a significant proportion of the targeted cells exhibit a defining feature of CCK-expressing basket cells.

### Subsets of targeted INs express NPY or PV

While our findings indicate that a substantial proportion of the targeted cells had properties of CCK/CB1R-expressing basket cells, two observations led us to wonder whether other IN populations were also targeted. First, light-evoked responses were not fully blocked by CB1R agonism, in contrast to earlier recordings obtained in BA (Vogel et al., 2016) and second, they displayed a prominent GABA_B_ receptor-mediated component in the light-evoked outward current that is uncharacteristic of basket cells (Vogel et al., 2016; Rovira-Esteban et al., 2017; Veres et al., 2017).

**Figure 3.**
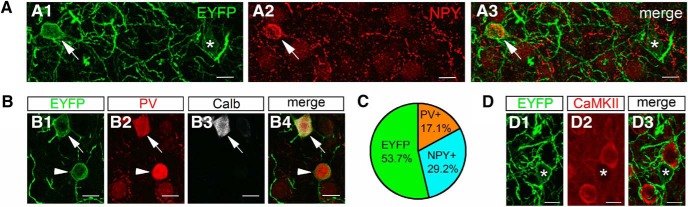
GABAergic cells labeled with intersectional viral strategy represent different populations of INs. ***A***, Example of EYFP-expressing cells (panel ***A1***) that either are (panel ***A2***, arrow) or are not (panel ***A3***, asterisk) also immunopositive for NPY (scale bar = 10 µm). ***B***, Example of two EYFP-expressing cells (panel ***B1***, arrow and arrowhead) that both contain PV (panel ***B2***), but only one of which is also immunopositive for Calb (panel ***B3***, arrow; scale bar = 10 µm). ***C***, Pie chart showing the ratio of EYFP-expressing neurons that contain PV or NPY. Note a large proportion of cells does not express either PV or NPY. ***D***, Example of EYFP-expressing cell (panel ***D1***, asterisk) and non-overlapping cells immunopositive for CaMKII (panels ***D2***, ***D3***; scale bar = 10 µm).

This led us to perform immunolabeling for presence of various IN neurochemical markers in EYFP-expressing cells. We found that ∼29% of EYFP+ cells expressed NPY (19 NPY+ out of 65 EYFP+ neurons) and ∼17% expressed PV (14 PV+ out of 82 EYFP+ neurons; Fig. 3*A–C*), whereas none of them expressed SOM (0 out of 33 EYFP+ neurons) or nNOS (0 out of 41 EYFP+ neurons; data not shown). Of note, PV and NPY only rarely co-expressed in the same neurons (1 PV+ neuron co-expressed NPY out of 174 PV+ neurons, while one NPY+ neuron contained PV out of 96 NPY+ neurons), consistent with prior studies (Mańko et al., 2012). We also detected Calb in some of PV/EYFP-expressing neurons (three of seven; Fig. 3*B*). Previous studies have established that Calb content of PV+ INs identifies the neuron as a basket cell (McDonald and Betette, 2001), as distinguished from PV+ INs lacking this Ca^2+^-binding protein, which are axo-axonic cells (Bienvenu et al., 2012; Vereczki et al., 2016; Andrási et al., 2017). Finally, in line with our *in situ* hybridization and electrophysiological data, none of the EYFP-expressing cells examined (*n* = 51) were immunopositive for a glutamatergic neuronal maker, CaMKII (Fig. 3*D*).

### Targeted INs are morphologically diverse

**Figure 4.**
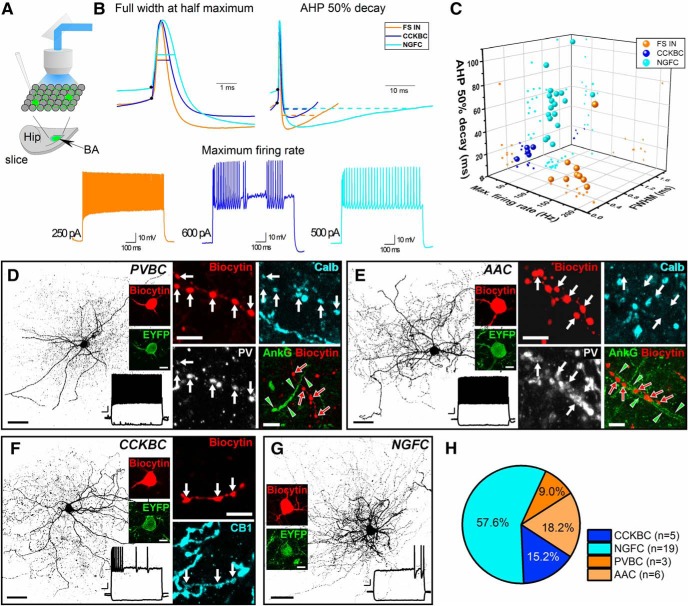
Action potential features distinguish GABAergic cell types labeled with intersectional viral strategy. ***A***, Schematic drawing depicts a horizontal slice with viral expression shown in green (Hip = hippocampus). Whole-cell patch clamp recordings were performed in INTRSECT-ChR2-transfected GABAergic cells (green circles) visualized by blue light illumination. ***B***, Traces exemplifying differences in the full width at action potential half maximum (FWHM), 50% decay of AHP and maximum firing rate for the three electrophysiologically distinct IN groups: fast-spiking INs (FS INs) in orange, CCK^+^ basket cells (CCKBCs) in blue, and NGFCs in cyan. ***C***, 3D plot showing the separation of 33 intracellularly labeled EYFP+ INs based on the three action potential parameters. ***D–G***, Examples of four distinct types of EYFP-expressing INs intracellularly filled by whole-cell recording *in vitro*. In each case, a maximal intensity projection of a 3D confocal image of the labeled INs is shown together with its firing pattern and the EYFP expression at the soma level. ***D***, An example for a PV+ basket cell (PVBC) identified based on its firing pattern, Calb and PV positivity in its axonal boutons (white arrows in insets) and forming no close appositions (red arrows) with ankyrin G (AnkG)-labeled axon initial segments (delimited by green arrowheads). ***E***, An example for a PV+ axo-axonic cell (AAC) identified based on its firing pattern, PV positivity and Calb negativity in its axonal boutons (white arrows in insets) and forming close appositions by its axonal boutons (red arrows) with an AnkG-labeled axon initial segment (delimited by green arrowheads). ***F***, An example for a CCKBC identified based on its firing pattern and on the CB1 content in its axonal boutons (white arrows in insets). ***G***, An example of a NGFC based on its dendritic and axonal morphology and characteristic firing pattern. ***H***, Pie chart showing the ratio of identified IN types in a group of EYFP-expressing neurons in the BA that were randomly sampled in slice preparations. For ***D–G*** depictions of maximal intensity projections of intracellularly filled cells, scale bar = 40 µm, insets = 5 µm; firing pattern scale bar *x*-axis = 100 ms, *y*-axis = 10 mV.

Our immunolabeling results suggest that, in addition to CCK/CB1R-expressing basket cells, three IN subtypes: PV+ basket cells, PV+ axo-axonic cells and NPY+, likely neurogliaform cells (NGFCs), were targeted. To substantiate this, we intracellularly-labeled and immunostained EYFP+ neurons obtained from our slice preparations to allow for a direct comparison between the firing properties and neurochemical phenotype of each cell (Fig. 4*A*). Of 33 EYFP+ cells labeled, all had dendritic and axonal morphologic features consistent with INs and not PNs. Examination of these cells based on the action potential width at half maximum, 50% decay time of the after-hyperpolarization (AHP) and maximum firing rate led us to classify three main subcategories which, in turn, corresponded well to differences in their respective immunocytochemically features (Fig. 4*B*).

One group was characterized by a fast-spiking phenotype (i.e., narrow action potential and high, >100-Hz firing-rate; Fig. 4*C*). Within this group, both PV+ basket cells (*n* = 3; Fig. 4*D*) and PV+ axo-axonic cells (*n* = 5; Fig. 4*E*) were identifiable; the former showed immunoreactivity for Calb and avoided the axon initial segments visualized by Ankyrin G staining, while the latter lacked Calb and their axonal varicosities formed close appositions with axon initial segments. A second group discharged action potentials with an intermediate width and at the lowest rate. The cells in this group had axonal varicosities immunopositive for CB1R, identifying them as CCK/CB1-expressing basket cells (*n* = 5; Fig. 4*F*). The third last group had the widest action potentials and longest AHP and were identifiable as NGFCs based on previously published results (Tamás et al., 2003; Armstrong et al., 2012; Mańko et al., 2012; *n* = 20; Fig. 4*G*). Indeed, quantification revealed the majority (∼60%) of the *in vitro* recorded and labeled cells fell into the latter, NGFC, subclass, with roughly equal (∼9–15%) proportions in the other classes (Fig. 4*H*).

### Photostimulating targeted INs facilitates fear extinction

**Figure 5.**
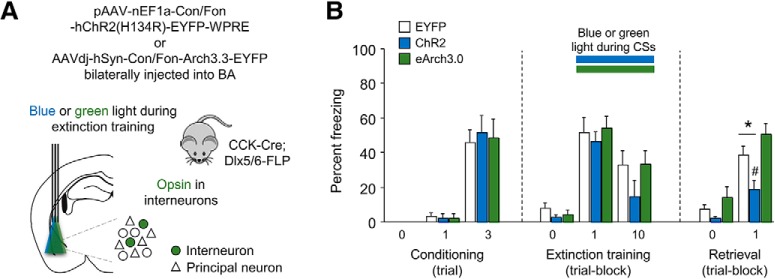
*In vivo* photostimulation of transfected INs during extinction training. ***A***, Schematic of the intersectional strategy used to target BA INs in CCK-Cre;Dlx5/6-Flp double-transgenic mice with INTRSECT pAAV-nEF1a-Con/Fon-hChR2(H134R)-EYFP-WPRE or AAVdj-hSyn-Con/Fon-Arch3.3-EYFP-WPRE virus and shine blue or green light during each CS presentation of extinction-training. For fiber placement maps see Extended Data Figure 5-1. ***B***, Photoexcitation, but not photoinhibition, during extinction training reduced freezing on light-free extinction retrieval the following day; *n* = 9–13 per group; *p < 0.05 INTRSECT-ChR2 versus INTRSECT-EYFP. #*p* < 0.05 T0 versus T1 extinction training for INTRSECT-ChR2.

Our next experiments assessed the contribution of the targeted population of BA INs to fear extinction. BA INs were infected with INTRSECT-ChR2 (Fig. 5*A*), INTRSECT-Arch, or an INTRSECT-EYFP control virus, and tested for fear conditioning, extinction training and extinction retrieval over consecutive days, using our standard extinction training protocol (Bukalo et al., 2015). For fiber placement maps see Extended Data Figure 5-1. During extinction training (only), blue or green light, respectively, was shone concomitant with each CS presentation.

10.1523/ENEURO.0220-19.2019.f5-1 Extended Data Figure 5-1 Virus expression and optic-fiber placement for CCK-Cre;Dlx5/6-FLP experiments. ***A***, Representative INTRSECT-ChR2 virus localization in the BA. ***B***, Cartoon depicting optic-fiber placements in the INTRSECT-ChR2 group. ***C***, Representative INTRSECT-Arch virus localization in the BA. ***D***, Cartoon depicting optic-fiber placements in the INTRSECT-Arch group. Download Figure 5-1, TIF file.

In all groups, freezing increased significantly from the first to third and final US-paired CS during conditioning (ANOVA effect of CS: *F*_(1,28)_ = 67.31, *p* < 0.001; effect of group: *F*_(2,28)_ = 0.07, *p* = 0.934; CS × group interaction: *F*_(2,28)_ = 0.13, *p* = 0.883; Fig. 5*B*). On extinction training, freezing decreased across CS trial-blocks (ANOVA effect of CS: *F*_(1,28)_ = 19.76, *p* < 0.001; effect of group: *F*_(2,28)_ = 0.99, *p* = 0.385; CS × group interaction: *F*_(2,28)_ = 0.58, *p* = 0.565; Fig. 5*B*).

During light-free extinction retrieval the following day, there was an overall decrease in freezing on extinction retrieval, as compared to the first trial-block of extinction training (ANOVA effect of CS: *F*_(1,28)_ = 9.24, *p* = 0.005; effect of group: *F*_(2,28)_ = 2.88, *p* = 0.073; CS × group interaction: *F*_(2,28)_ = 1.90, *p* = 0.169; *post hoc* paired *t* test retrieval vs training-trial comparisons for EYFP: *t*_(12)_ = 1.81, *p* = 0.095; ChR2: *t*_(8)_ = 3.20, *p* = 0.013, eArch3.3: *t*_(8)_ = 0.37, *p* = 0.719). The INTRSECT-ChR2 group exhibited significantly less freezing than INTRSECT-EYFP controls, or the INTRSECT-eArch group (effect of group: *F*_(2,28)_ = 7.29, *p* = 0.003; *post hoc* comparisons for EYFP vs ChR2: *p* = 0.043; ChR2 vs eArch: *p* < 0.001; Fig. 5*B*), consistent with the facilitation of extinction memory formation.

### Non-selectively photostimulating or photosilencing BA CCK cells disrupts fear extinction

**Figure 6.**
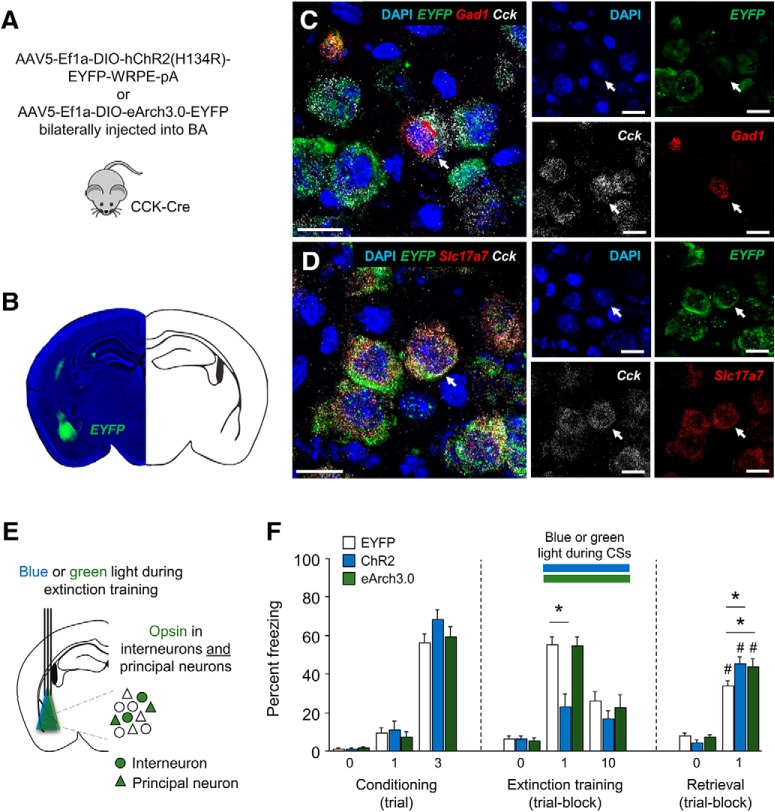
*In vivo* photostimulation and photosilencing of transfected BA INs and PNs during extinction training. ***A***, Schematic of approach to targeting BA INs and PNs in CCK-Cre single-transgenic mice with AAV5-Ef1a-DIO-hChR2(H134R)-EYFP-WRPE-pA or AAV5-Ef1a-DIO-eArch3.0-EYFP. ***B***, Representative example of EYFP expression after virus transfection. ***C***, Virus-transfected (EYFP-expressing) cells labeled with *Cck* mRNA, including example of cell labeled with *Gad1* mRNA (white arrows, scale bar = 20 µm). ***D***, Virus-transfected (EYFP-expressing) cells labeled with *Cck* mRNA, including example of cell labeled with *Slc17a7* mRNA (white arrows, scale bar = 20 µm). ***E***, Blue (ChR2 group) or green (eArch3.0 group) was shone during each CS presentation of extinction training. For fiber placement maps see Extended Data Figure 6-1. ***F***, Photoexcitation in the ChR2 group during each CS presentation of extinction training increased freezing during training and light-free extinction retrieval the following day, relative to EYFP controls. Photosilencing in the eArch3.0 group during each CS presentation of extinction training reduced freezing on light-free extinction retrieval the following day, relative to EYFP controls; *n* = 12–27 per group; *p < 0.05, #*p* < 0.05 T0 versus T1 extinction training. Results of similar interventions on locomotion and anxiety level are presented in Extended Data Figure 6-2.

Given the observation of an extinction-facilitating effect of photostimulating BA INs, we wondered how this effect would compare with the effect of manipulating a combination of BA INs and BA CCK-expressing glutamatergic neurons. We transfected cells of single-transgenic CCK-Cre mice in the BA with Cre-dependent ChR2, eArch3.0 or an EYFP control virus (Fig. 6*A*,*B*). Given the majority of amygdalar glutamatergic neurons express CCK (Allen Brain Institute, experiment: 77869074), we expected the effect of non-selectively photostimulating BA CCK cells would largely reflect stimulation of glutamatergic cells and thereby differ from the effect of BA INs. Using fluorescence *in situ* hybridization we found that following virus injections into the BA, EYFP-labeled neurons were positive for *Cck* mRNA and that some of these cells showed labeling for *Gad1* and other for *Slc17a7*, consistent with the transfection of both CCK INs and PNs (Fig. 6*C*,*D*; Andrási et al., 2017).

10.1523/ENEURO.0220-19.2019.f6-1 Extended Data Figure 6-1 Virus expression and optic-fiber placements for CCK-Cre experiments. ***A***, Representative ChR2 virus localization in the BA. ***B***, Cartoon depicting optic-fiber placements in the ChR2 group. ***C***, Representative eArch3.0 virus localization in the BA. ***D***, Cartoon depicting optic-fiber placements in the eArch3.0 group. Download Figure 6-1, TIF file.

10.1523/ENEURO.0220-19.2019.f6-2 Extended Data Figure 6-2 *In vivo* photostimulation and photosilencing of transfected BA INs and PNs during a novel open field test. ***A***, Neither photoexcitation in the ChR2 group nor photosilencing in the eArch3.0 group altered total distance traveled, relative to EYFP controls. ***B***, Neither photoexcitation in the ChR2 group nor photosilencing in the eArch3.0 group altered center zone distance traveled, relative to EYFP controls; *n* = 6–17 per group. Download Figure 6-2, TIF file.

We next performed fear conditioning and extinction testing (as above). For fiber placement maps see Extended Data Figure 6-1.

Freezing was significantly higher on the third CS than the first CS during fear conditioning, with no difference between groups (ANOVA effect of CS: *F*_(1,52)_ = 182.05, *p* < 0.001; effect of group: *F*_(2,52)_ = 1.26, *p* = 0.29; CS × group interaction: *F*_(2,52)_ = 0.61, *p* = 0.55; Fig. 6*F*). On extinction training, freezing decreased across CS trial-blocks in the EYFP and eArch3.0 groups and the ChR2 group froze less on the first trial-block than EYFP controls (ANOVA effect of CS: *F*_(1,52)_ = 27.71, *p* < 0.001; effect of group: *F*_(2,52)_ = 6.74, *p* = 0.003; CS × group interaction: *F*_(2,52)_ = 3.11, *p* = 0.053, followed by *post hoc* comparison of ChR2 vs EYFP on the first trial-block: *p* < 0.001; last trial-block; Fig. 6*F*).

There was a decrease in freezing on extinction retrieval, as compared to the first trial of extinction training, in all groups (ANOVA effect of CS: *F*_(1,52)_ = 1.09, *p* = 0.302; effect of group: *F*_(2,52)_ = 3.72, *p* = 0.031; CS × group interaction: *F*_(2,52)_ = 17.37, *p* < 0.001; *post hoc* paired *t* test retrieval vs training-trial comparisons for EYFP: *t*_(26)_ = 5.05, *p* < 0.001; ChR2: *t*_(11)_ = 2.87, *p* = 0.015, eArch3.0: *t*_(15)_ = 2.88, *p* = 0.011). However, freezing differed significantly between groups on the subsequent extinction retrieval test, with higher freezing in both the ChR2 and eArch3.0 group, as compared to the EYFP controls (effect of group: *F*_(2,52)_ = 3.79, *p* = 0.029; *post hoc* comparisons for EYFP vs ChR2: *p* = 0.026; EYFP vs eArch3.0 *p* = 0.031; Fig. 6*F*), indicating an impairment in fear extinction in response to either silencing or excitation of this neuronal population.

Finally, we used a novel open field test to confirm that the differences observed in the extinction retrieval were due to the manipulation of the BA CCK^+^ cells participating in fear circuits, and not to a different locomotor or anxiety level within the different groups. In this test, groups did not differ in anxiety-related exploration of the aversive center zone (effect of group: *F*_(2,43)_ = 8.098, *p* = 0.001) or total distance traveled in the arena (effect of group: *F*_(2,43)_ = 8.098, *p* = 0.001; Extended Data Fig. 6-2).

## Discussion

There is growing appreciation of the critical contribution of INs to the regulation of network activity to support behavioral functions subserved by the BA, including Pavlovian fear and extinction. However, a description of the subclass of BA INs expressing the neuropeptide, CCK, has proved elusive. Here, we sought to genetically access BA CCK INs to define the neurochemical and physiologic phenotype of these cells and assess their possible contribution to fear extinction.

Using an intersectional approach, entailing transfecting Cre+/Flp+ cells in CCK-Cre;Dlx5/6-Flpe transgenic mice with opsin-containing INTRSECT viruses, we selectively targeted GABAergic INs in the BA and showed that almost half of these expressed functional CB1R on their axonal boutons. In line with this anatomic observation, the application of a CB1R agonist reduced the amplitude of light-evoked IPSCs by half. Taken together, this is strong evidence that a significant portion of the cells targeted by INTRSECT strategy are CCK-expressing basket cells, given prior studies have shown that CCK-expressing basket cells express CB1R on their boutons that, when activated, markedly suppress inhibitory transmission (Katona et al., 2001; McDonald and Mascagni, 2001a; Azad et al., 2004; Vogel et al., 2016; Barsy et al., 2017; Rovira-Esteban et al., 2017; Veres et al., 2017). This functional connection between CCK and CB1R has been of particular interest to the field given compelling evidence linking endocannabinoid signaling, in the BA and other regions, to extinction (Patel et al., 2017). One proposal is that endocannabinoids could inhibit CCK release in the BA (Beinfeld and Connolly, 2001; Chhatwal et al., 2009) and thereby oppose the peptide’s pro-fear/anxiety effects to enable extinction (Frankland et al., 1997; Harro, 2006; Del Boca et al., 2012; Bowers and Ressler, 2015).

We found that the genetically targeted INs also displayed a prominent GABA_B_ receptor-mediated component in the light-evoked outward current that likely does not originate from CCK-expressing basket cells. Indeed, further examination using a combination of immunostaining, electrophysiological recordings and morphologic inspection indicated that targeting CCK cells also included PV+ basket cells, PV+ axo-axonic cells, and NGFCs. A portion of the latter INs might express NPY (Mańko et al., 2012). Based on available data, we conclude that recombination in these cells reflects the genuine presence of CCK in adult BA cells at low levels that were not detected by prior studies using other approaches. This conclusion is based on several observations. First, other recent studies using genetic approaches also support a significant diversity of CCK-expressing INs in the BA, among which INs with NGFC morphology and firing characteristics were evident (Jasnow et al., 2009; Vogel et al., 2016; Veres et al., 2017). Second, CCK mRNA was unequivocally detected in axo-axonic cells both in the neocortex (Paul et al., 2017) and hippocampus (Harris et al., 2018) and even in a portion of hippocampal PV-expressing basket cells (Harris et al., 2018). Third, using a different approach to that used herein, involving crossing offspring of CCK-Cre and Nkx2.1-Flp mice with an Ai65 reporter line, another recent study reported labeling of axo-axonic cells in the neocortex (Paul et al., 2017). Fourth, dense core vesicles are readily observed in axon terminals of both PV-containing basket and axo-axonic cells (Takács et al., 2015), indicating the presence of neuropeptides in these GABAergic cell types that have not been labeled so far in SOM-Cre, NPY-Cre, or VIP-Cre mouse lines. Taken together with these prior observations, our results strongly speak to the importance of applying multiple phenotypic criteria when classifying CCK IN cells and underscore the limitations of demarcating this population based on a single, neurochemical marker (Harris et al., 2018). The difference in ratio of distinct IN types obtained by immunostaining in perfused tissue (Figs. 2, 3) and by randomly sampled neurons recorded in *in vitro* slices (Fig. 4) may reflect the fact that some cell types are better able to tolerate the procedure of slice preparation than others.

The finding that optogenetic photostimulation of the targeted IN population in the BA produced behavioral effects is indicative of a facilitation of fear extinction. This is reminiscent of the recent finding that brain-wide chemogenetic activation of a population of INs genetically-accessed using a CCK-Cre;Dlx5/6-Flpe transgenic strategy similar to ours, improved performance on memory tasks (contextual fear and discrimination, social and object recognition, puzzle box; Whissell et al., 2019). Together, these behavioral effects raise intriguing questions about the relative contribution of specific subsets of INs that are targeted by this strategy, given our data show that the population are not simply “CCK-expressing,” at least assessed by immunostaining. Earlier studies have implicated BA INs including NPY-expressing and PV-expressing cells in extinction (Gutman et al., 2008; Herry et al., 2010; Verma et al., 2012; Tovote et al., 2015). For instance, a reduction in GABAergic input from PV-expressing axo-axonic cells onto BA PNs resulted in an impaired extinction learning (Saha et al., 2017). However, whether one specific subset disproportionally contributes to the extinction-facilitating effects of stimulation remains to be determined and will be technically challenging to address, given the lack of exclusive markers for each given subpopulation. Another important goal will be positioning the various subclasses into the micro and macro circuits mediating extinction. Prior work has already demonstrated important functional connections both locally between different IN subtypes in the BA (Andrási et al., 2017) and distally, via CCK IN projections to the medial PFC regions (Senn et al., 2014). In summary, our data together with recent findings imply that excitation of GABAergic microcircuits in the BA via local or distal projections with cortical or subcortical origin could potentially augment extinction memory formation.

In experiments using single CCK-Cre mice, we targeted a substantial portion of PNs (in addition to CCK INs) located in the BA, reflecting the fact that most excitatory neurons express CCK in this nucleus (Rovira-Esteban et al., 2017; see also Allen Brain Atlas, experiment: 77869074). In contrast to the extinction-facilitating effects of photostimulating the IN population, photostimulation or photosilencing of globally-targeted (i.e., INs and PNs) BA CCK cells, via Cre-dependent opsin transfection in CCK-Cre single-transgenic mice, during extinction training led to an impairment in long-term extinction memory, as evidenced by higher freezing on a light-free extinction retrieval test. Of note, photostimulation also reduced freezing during initial extinction training (i.e., fear retrieval), which suggests either an acute anxiolytic-like effect or a failure to retrieve the fear memory. Alternatively, this reduction in freezing could reflect a photostimulation-induced potentiation of CS-induced escape behaviors, which would be in line with panicogenic effects of CCK agonism (de Montigny, 1989; Bradwejn et al., 1991; Kramer et al., 1995). This interpretation awaits further testing although we did not detect effects of photostimulation on motor or anxiety-related behaviors in a novel open field test in which no CS was presented.

Our data obtained in single CCK-Cre mice showing that photoinhibition of BA neurons impaired fear retrieval are in line with recent findings using a distinct mouse line, Thy1-Cre. As in our case, photoinhibition of Thy1-expressing, mainly PNs in the BA during extinction training resulted in a weakening in extinction memory formation (McCullough et al., 2016). These results suggest that BA circuits contain neural populations able to control fear extinction memory (Herry et al., 2008).

Impairments in fear extinction are evident in various neuropsychiatric conditions, including trauma- and stressor-related disorders and some anxiety disorders. This has encouraged basic researchers to define the neurobiological basis of impaired and intact fear extinction as a potentially tractable approach to developing new treatments for these disorders. The resultant research has defined the amygdala as a central node within a distributed neural system comprising cortical, hippocampal, and midbrain structures, among others. The current findings add to a growing literature by describing a unique population of INs in the BA that, when activated, exert strong modulatory effects on extinction.

## Synthesis

Reviewing Editor: Katalin Toth, Universite Laval Decisions are customarily a result of the Reviewing Editor and the peer reviewers coming together and discussing their recommendations until a consensus is reached. When revisions are invited, a fact-based synthesis statement explaining their decision and outlining what is needed to prepare a revision will be listed below. The following reviewer(s) agreed to reveal their identity: Marco Capogna, Kaspar Vogt.

This is an original study with novel information on the CCK-positive interneuron population the BLA and their role in fear extinction. The work is high quality and well organized.

The most critical comment that the authors would need to address came from Rev.#2. he/she raised a valid question about the specificity of the expression patterns, and rightly argued that the work is hinges on this issue. The authors must provide a detailed explanation and provide all available data to strengthen their arguments. Beside this critical point, both reviewers raised other smaller issues, that the authors need to carefully address.

Rev.#1.

This manuscript eN-NWR-0220-19 shows original and unprecedented information on cholecystokinin-expressing interneurons of rodent basolateral amygdala. The Authors observed that four interneuron types belong to this group of cells: CB1+ basket cell, neurogliaform cell, parvalbumin-expressing basket cell and axo-axonic cell. They also provide information on CCK+ cells' role on fear memory extinction. The manuscript contains mostly convincing and high quality data; ample and satisfactory technical information is given on the various techniques used in the method section; data are appropriately analyzed and illustrated. Particularly, all the data related to CCK+ interneurons are entirely convincing. A minor comment for this part is that the Authors should better highlight what pattern of optogenetic stimulation or inhibition they used during the in vitro and in vivo experiments and provide clear rationales behind their choices. Furthermore, the data regarding general manipulation of BA CCK-expressing neurons require some more attention. Specifically:

1) The Authors should provide a better rationale to justify the inclusion of results shown in Fig 6, than the one presently advanced (to reproduce the extinction-facilitating effect of CCK+ IN photostimulation). It is expected that to stimulate simultaneously excitatory and inhibitory neurons may produce somehow puzzling results.

2) It would be useful to include more information on CCK-expressing non-GABAergic cells. For example, are these pyramidal cells with the soma in the lateral amygdala, basolateral amygdala or both? Is it known where these cells project? How much they send/receive inputs from CCK+ interneurons? Also, provide better explanation (or at least include a reference) why the labelling with Slc17a7 is specific to pyramidal cells.

Rev.#2.

This is an extensive study of BLA CCK+ IN function. It reveals a surprising functional and anatomical diversity, not previously described. As stated above the autors do have to be sure that their targeting stratey works, their discussion section is not reassuring in its current state

The INTRSECT strategy in restricting the expression of optogenetic tools to CCK interneurons is useful and if it is really working, valuable. The behavioral data with a clear phenotype in fear extinction is an important addition to our understanding of CCK+ IN function in the BLA.

Two main issues:

1.) The quality of the work absolutely hinges on the specificity of the expression patterns. The subtypes of IN that showed expression is unusual, as the authors themselves acknowldege. However, the explanation in the discussion is somewhat alarming. “Recombination in these cells likely might reflect the genuine presence of CCK in these IN populations. either transiently during development and/or in adult BA at low levels that were difficult to detect by prior studies using other approaches”. It should not be “likely might” - either these neurons express the vector based on the GABAergic and CCK+ phenotype or they don't. If they don't the results become very difficult to interpret, since we are then dealing with an ill-described mixture of INs. The authors also state “Such technical issues speak to the limitation of demarcating CCK IN population based on a single neurochemical marker...”. There are two separate issues here and they are unfortunately conflated. One is the specificity of their targeting (see above) and the other is the heterogeneity of the (properly!) targeted CCK+ population. The first is a technical issue that needs to be solved and the second a potentially interesting feature of BLA CCK+ IN.

I am unsure how developmental variability in CCK expression could be a factor here, since AAV mediated transfection was used to express the optogenetic tools. Can the authors expalin this more clearly?

Finally, the authors should state unequivocally whether they trust ther system to specifically and selectivelly label CCK+ BLA GABAergic INs and what this trust is based on.

2) The entire discussion of potential effects of CCK release istelf is not supported by the data presented and entirely speculative. The fact that non-selective targeting of CCK+ neurons does not replicate the phenotype obtained by CCK+ IN targeting would argue against a CCK effect, together with the fact that both stimulating and inhibiting the CCK+ neuronal population lead to the same behavioral outcome.

According to the authors CCK release and CCK-B-R activation are anxiogenic, but activation of potentially CCK releasing INs increases fear extinction acquisition and does not produce more freezing. What is the author's explanation for this finding? It also seems somewhat contradictory to the finding that CB1 activation is anxiolytic given the fact that CB1 activation depresses synaptic release. Can the authors discuss this apparent contradiction?

It would be generally useful for the authors to discuss their circuit model of BLA IN function in fear extinction acqusition in the light of their findings.

A few minor points:

Do the authors really mean that photostimulation (either activating or depressing!?) of the CCK+ neuronal population “impaired subsequent (light free) retrieval”. Fig 6 F indicates that retrieval is enhanced (freezing increased) in the stimulated group. Am I missing something?

Figure 1 E: Is the EYFP reactivity really restricted to the two marked neurons (arrow)? What were the cutoff criteria used?

Figure 2 J: What is the reason for the discrepancy in number of traces used for peak amplitude comparison of fast and slow (23) and charge transfer of fast and slow (10)?

If CB1 receptor expression is a defining feature of CCK positive neurons, what is the explanation for the relatively low expression levels found (40%)?

Figure 3 D: How were EYFP positive baskets formed around CaMKII positive neurons distinguished from expression of EYFP in CaMKII positive neurons. The figure is not that clear in this regard.

Figures 5-1 6-1 and 6B are difficult to decipher - the dark blue label seems not to serve any clear purpose.

## Author Response

Rev.#1

“The manuscript contains mostly convincing and high quality data; ample and satisfactory technical information is given on the various techniques used in the method section; data are appropriately analyzed and illustrated. Particularly, all the data related to CCK+ interneurons are entirely convincing.”

Response: We are grateful to the Reviewer for acknowledging the quality of these data and the multiple technical approaches employed.

1) The Authors should provide a better rationale to justify the inclusion of results shown in Fig 6, than the one presently advanced (to reproduce the extinction-facilitating effect of CCK+ IN photostimulation). It is expected that to stimulate simultaneously excitatory and inhibitory neurons may produce somehow puzzling results.

Response: We agree that clear a priori predictions regarding the effects of these manipulations are difficult, for the reason the Reviewer points outs, and have re-phrased the rationale for performing the experiments shown in Fig. 6. 'Given the observation of an extinction-facilitating effect of photostimulating BA INs, we wondered how this effect would compare with the effect of manipulating a combination of BA INs and BA CCK-expressing glutamatergic neurons. We transfected cells of single-transgenic CCK-Cre mice in the BA with Cre-dependent ChR2, eArch3.0 or an EYFP control virus (Figure 6A, B). Given the majority of amygdalar glutamatergic neurons express CCK (Allen Brain Institute, Experiment: 77869074), we expected the effect of non-selectively photostimulating BA CCK cells would largely reflect stimulation of glutamatergic cells and thereby differ from the effect of BA INs..'

2) It would be useful to include more information on CCK-expressing non-GABAergic cells. For example, are these pyramidal cells with the soma in the lateral amygdala, basolateral amygdala or both? Is it known where these cells project? How much they send/receive inputs from CCK+ interneurons? Also, provide better explanation (or at least include a reference) why the labelling with Slc17a7 is specific to pyramidal cells.

Response: Regarding LA expression: CCK is also expressed in LA principal neurons, but, as shown in our representative images, our injections targeted the BA, where the vast majority, although not all the labelled neurons were located. Therefore, the contribution of the function of LA neurons to our findings should be minimal.

Regarding projection and interneuron interconnectivity: Based on the Reviewer's questions, we have added to the Discussion as follows: 'In experiments using single CCK-Cre mice, we targeted a substantial portion of principal neurons (in addition to CCK INs) located in the BA, reflecting the facts that most excitatory neurons express CCK in this nucleus (Rovira-Esteban et al., 2017, see also Allen Brain Atlas, Experiment: 77869074).' To our knowledge, no data are available for the input-output features of CCK-expressing principal neurons in the amygdala, and this is an interesting avenue for future work.

Regarding the use of Slc17a7 as a label: We included a reference supporting the selectively of Slc17a7 as a maker for amygdalar principal cells, based on the overlapping expression of Slc17a7 labelling with Cre-driven Slc17a7 (Andrasi et al., 2017). In contrast, in amygdala and other cortical regions, CaMKII, which is often used as a marker for principal neurons, is expressed in glutamatergic principal cells but also in some interneurons, including fast spiking parvalbumin-containing GABAergic cells (Watakabe et al., 2015). For this reason, we used Slc17a7 rather than CaMKII to reveal the glutamatergic phenotype of neurons.

3) A minor comment for this part is that the Authors should better highlight what pattern of optogenetic stimulation or inhibition they used during the in vitro and in vivo experiments and provide clear rationales behind their choices.

Response: We have added explanation to the methods, as follows: 'For photostimulation, blue light (λ = 473 nm) was bilaterally shone on the BA at 20 Hz, in 5-ms pulses, consistent with commonly used ChR2-excitation parameters and mimicking the activity of highly active neurons. For photosilencing, green light (λ = 532 nm) was bilaterally shone on the BA continuously; which is also in line with commonly used Arch-excitation parameters.'

4) Furthermore, the data regarding general manipulation of BA CCK-expressing neurons require some more attention.

Response: As noted in the Response to point 2, we have embellished a little on those data.

Rev.#2

The behavioral data with a clear phenotype in fear extinction is an important addition to our understanding of CCK+ IN function in the BLA.

Response: We thank the Reviewer for recognizing what we also think could be an important addition to the literature.

1) The quality of the work absolutely hinges on the specificity of the expression patterns. The subtypes of IN that showed expression is unusual, as the authors themselves acknowldege. However, the explanation in the discussion is somewhat alarming. “Recombination in these cells likely might reflect the genuine presence of CCK in these IN populations. either transiently during development and/or in adult BA at low levels that were difficult to detect by prior studies using other approaches”. It should not be “likely might” - either these neurons express the vector based on the GABAergic and CCK+ phenotype or they don't. If they don't the results become very difficult to interpret, since we are then dealing with an ill-described mixture of INs. The authors also state “Such technical issues speak to the limitation of demarcating CCK IN population based on a single neurochemical marker...”. There are two separate issues here and they are unfortunately conflated. One is the specificity of their targeting (see above) and the other is the heterogeneity of the (properly!) targeted CCK+ population. The first is a technical issue that needs to be solved and the second a potentially interesting feature of BLA CCK+ IN. I am unsure how developmental variability in CCK expression could be a factor here, since AAV mediated transfection was used to express the optogenetic tools. Can the authors expalin this more clearly? Finally, the authors should state unequivocally whether they trust ther system to specifically and selectivelly label CCK+ BLA GABAergic INs and what this trust is based on.

Response: We agree these are key points and have modified the Discussion as follows: 'Based on available data, we conclude that recombination in these cells reflects the genuine presence of CCK in adult BA cells at low levels that were not detected by prior studies using other approaches. This conclusion is based on several observations. First, other recent studies using genetic approaches also support a significant diversity of CCK-expressing INs in the BA, among which interneurons with NGFC morphology and firing characteristics were evident (Jasnow et al., 2009; Vogel et al., 2016; Veres et al., 2017). Second, CCK mRNA was unequivocally detected in axo-axonic cells both in the neocortex (Paul et al., 2017) and hippocampus (Harris et al., 2018) and even in a portion of hippocampal PV-expressing basket cells (Harris et al., 2018). Third, using a different approach to that used herein, involving crossing offspring of CCK-Cre and Nkx2.1-Flp mice with an Ai65 reporter line, another recent study reported labeling of axo-axonic cells in the neocortex (Paul et al., 2017). Fourth, dense core vesicles are readily observed in axon terminals of both PV-containing basket and axo-axonic cells (Takacs et al., 2015), indicating the presence of neuropeptides in these GABAergic cell types that have not been labelled so far in SOM-Cre, NPY-Cre or VIP-Cre mouse lines. Taken together with these prior observations, our results strongly speak to the importance of applying multiple phenotypic criteria when classifying CCK IN cells and underscore the limitations of demarcating this population based on a single, neurochemical marker (Harris et al., 2018).'

2) The entire discussion of potential effects of CCK release istelf is not supported by the data presented and entirely speculative. The fact that non-selective targeting of CCK+ neurons does not replicate the phenotype obtained by CCK+ IN targeting would argue against a CCK effect, together with the fact that both stimulating and inhibiting the CCK+ neuronal population lead to the same behavioral outcome.

According to the authors CCK release and CCK-B-R activation are anxiogenic, but activation of potentially CCK releasing INs increases fear extinction acquisition and does not produce more freezing. What is the author's explanation for this finding? It also seems somewhat contradictory to the finding that CB1 activation is anxiolytic given the fact that CB1 activation depresses synaptic release. Can the authors discuss this apparent contradiction? It would be generally useful for the authors to discuss their circuit model of BLA IN function in fear extinction acqusition in the light of their findings.

Response: Based on the Reviewer's suggestion, we have omitted this part of the Discussion and extended it with a circuit-based view, as follows: 'For instance, a reduction in GABAergic input from PV-expressing axo-axonic cells onto BA principal neurons resulted in an impaired extinction learning (Saha et al., 2017).' And 'In summary, our data together with recent findings imply that excitation of GABAergic microcircuits in the BA via local or distal projections with cortical or subcortical origin could potentially augment extinction memory formation. ' And 'Our data obtained in single CCK-Cre mice showing that photoinhibition of BA neurons impaired fear retrieval is in line with recent findings using a distinct mouse line, Thy1-Cre. As in our case, photoinhibition of Thy1-expressing, mainly principal neurons in the BA during extinction training resulted in a weakening in extinction memory formation (McCullough et al., 2016). These results suggest that BA circuits contain neural populations able to control fear extinction memory (Herry et al., 2008).'

3) Do the authors really mean that photostimulation (either activating or depressing!?) of the CCK+ neuronal population “impaired subsequent (light free) retrieval”. Fig 6 F indicates that retrieval is enhanced (freezing increased) in the stimulated group. Am I missing something?

Response: The reviewer is correct that both manipulations produced an effect in the same direction. We have rephrased the description of this effect to be clearer, as follows: 'In contrast to the extinction-facilitating effects of photostimulating the IN population, photostimulation or photosilencing of globally-targeted (i.e., INs and PNs) BA CCK cells - via Cre-dependent opsin transfection in CCK-Cre single-transgenic mice - during extinction training led to an impairment in long-term extinction memory, as evidenced by higher freezing on a light-free extinction retrieval test.'

4) Figure 1 E: Is the EYFP reactivity really restricted to the two marked neurons (arrow)? What were the cutoff criteria used?

Response: In this image, which is representative of the pattern of expression evident in these mice, the arrows denote two neurons that are positive for EYFP, Cck and Gad1, not just EYFP. This is now clarified in the legends, as follows: “(E) Virus transfected (EYFP-expressing) cells labeled with Cck and Gad1 mRNA; arrows denote 2 example neurons positive for EYFP, Cck and Gad1(scale bar 10 = µm).” These are determinations based on visual inspection of the image rather than a formal quantitative cutoff.

5) Figure 2 J: What is the reason for the discrepancy in number of traces used for peak amplitude comparison of fast and slow (23) and charge transfer of fast and slow (10)?

Response: In all recordings (n=23) we could measure the peak amplitude of both components, however, the area of fast and slow events was calculated only after applying Gabazine (n=10) and subtracting the remaining current area (slow component) from the original light-evoked composed current. This is now described in the methods, as follows: “The peak amplitude for the fast and slow components was determined at distinct time points (n=23; Figure 2E). The area of these two components was calculated only for those events in which the fast component was blocked by Gabazine (n=10): the remaining slow component was subtracted from the original trace resulting in the area for the fast component.”

6) If CB1 receptor expression is a defining feature of CCK positive neurons, what is the explanation for the relatively low expression levels found (40%)?

Response: CB1R is a defining feature of CCK-expressing basket cells that compose only a part of all CCK+ INs based on others' and our observations. To be more explicit, we modified the results section: 'Based on earlier reports that CCK-containing basket cells in the BA express the cannabinoid CB1R subtype, we next asked whether the targeted BA INs were CB1R-positive (Katona et al., 2001; McDonald and Mascagni, 2001a; Vereczki et al., 2016; Veres et al., 2017). To test the responsivity of the targeted cells to CB1R activation, we generated light-evoked postsynaptic currents in EYFP-negative cells followed by bath application of the CB1R agonist, CP 55,940. The application of the agonist reduced the peak amplitude of light-evoked events by 50% (control: 209.8 {plus minus} 88.4 pA, in CP: 112.6 {plus minus} 47.6 pA, peak ratio: CP/ctr: 51.3 {plus minus} 5.4 %, n = 5, t(4) = 2.35, P = 0.039) (Figure 2K-L). We verified that the CP 55,940-induced reduction was CB1R-mediated by abolishing the effect via pre-incubation with the CB1R antagonist, AM251 (peak amplitude in AM251: 261.8 {plus minus} 70.2 pA, in AM251 + CP 55,940: 259. 5 {plus minus} 78.4 pA, peak ratio: AM251 + CP 55,9940/AM251: 96.7 {plus minus} 8.9 %, n = 4, P = 0.89; Figure 2M, N). These data demonstrate that a significant component of synaptic currents in BA neurons evoked by light illumination in slices stems from signaling through the CB1R. Consistent with these finding, immunostaining BA sections containing transfected processes revealed that ~40% of EYFP-expressing axonal varicosities were positive for CB1R (Figure 2O-R).'

7) Figure 3 D: How were EYFP positive baskets formed around CaMKII positive neurons distinguished from expression of EYFP in CaMKII positive neurons. The figure is not that clear in this regard.

Response: As EYFP is expressed not only in the cell membrane but also in the cytoplasm, we consider neurons to be EYFP+ if their cytoplasm in addition to the cell membrane showed immunoreactivity, which was not the case for those cells that were immunopositive for CaMKII. This is now clarified in the methods, as follows: “Those neurons that showed EYFP immunoreactivity in both the cytoplasm and cell membrane were considered EYFP+ and could be clearly distinguished from those EYFP- neurons that soma and proximal dendrites were surrounded by axon varicosities expressing EYFP (Figure 3).”

8) Figures 5-1 6-1 and 6B are difficult to decipher - the dark blue label seems not to serve any clear purpose.

Response: The dark blue label denotes a DAPI stain that is commonly used to visualize neurons. We included the DAPI stain in these images to help the reader visualize the total number of neurons in the section.
