# Supplementary figures and images for: Excitation of Diverse Classes of Cholecystokinin Interneurons in the Basal Amygdala Facilitates Fear Extinction
Source: eNeuro. 2019 Nov 6;6(6):ENEURO.0220-19.2019. doi: 10.1523/ENEURO.0220-19.2019 (PMC6838687; doi:10.1523/ENEURO.0220-19.2019)

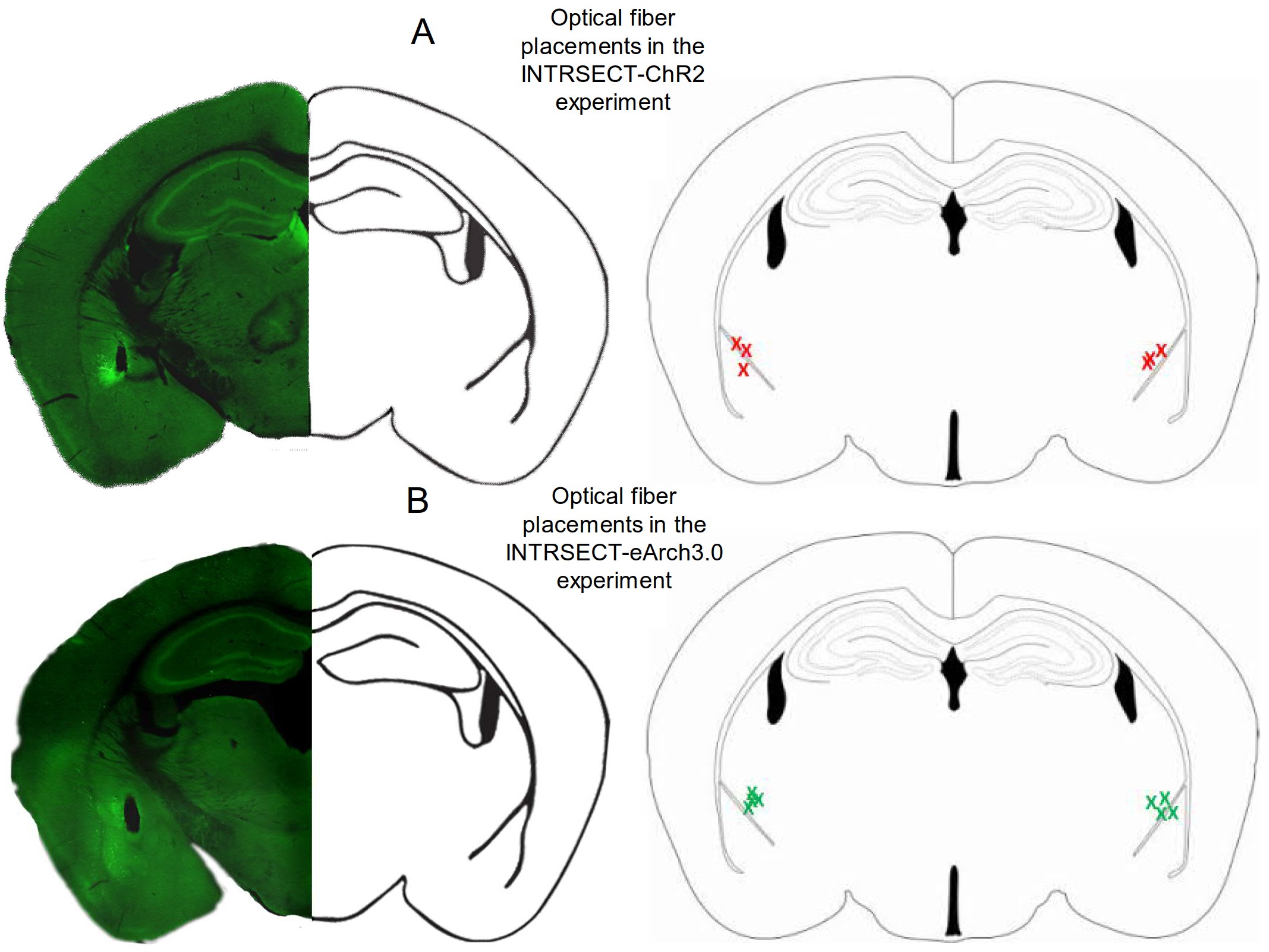

Supplement: Extended Data Figure 5-1 — Virus expression and optic-fiber placement for CCK-Cre;Dlx5/6-FLP experiments. A, Representative INTRSECT-ChR2 virus localization in the BA. B, Cartoon depicting optic-fiber placements in the INTRSECT-ChR2 group. C, Representative INTRSECT-Arch virus localization in the BA. D, Cartoon depicting optic-fiber placements in the INTRSECT-Arch group. Download Figure 5-1, TIF file. [file sup_enu-eN-NWR-0220-19-s01.tif]

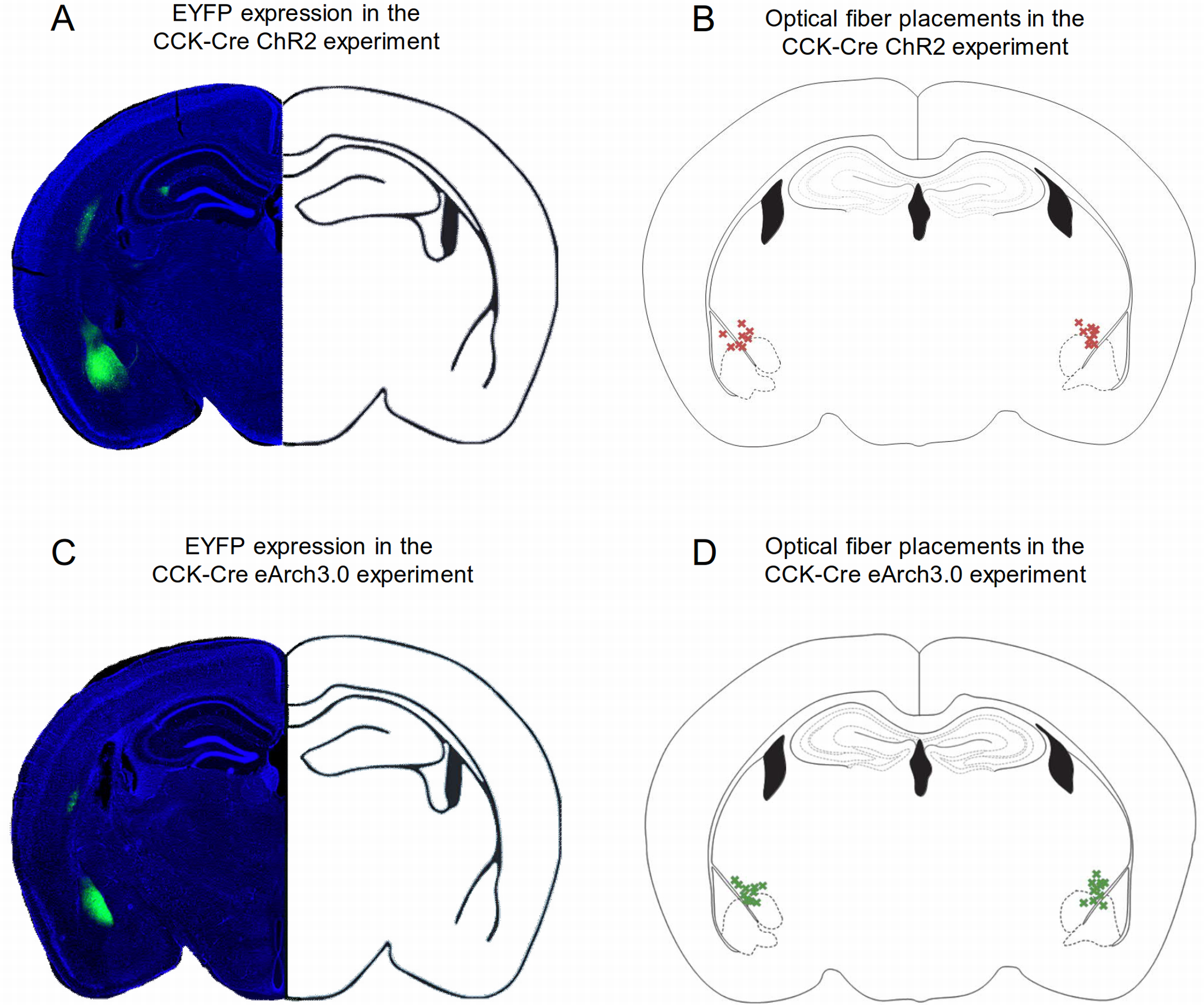

Supplement: Extended Data Figure 6-1 — Virus expression and optic-fiber placements for CCK-Cre experiments. A, Representative ChR2 virus localization in the BA. B, Cartoon depicting optic-fiber placements in the ChR2 group. C, Representative eArch3.0 virus localization in the BA. D, Cartoon depicting optic-fiber placements in the eArch3.0 group. Download Figure 6-1, TIF file. [file sup_enu-eN-NWR-0220-19-s02.tif]

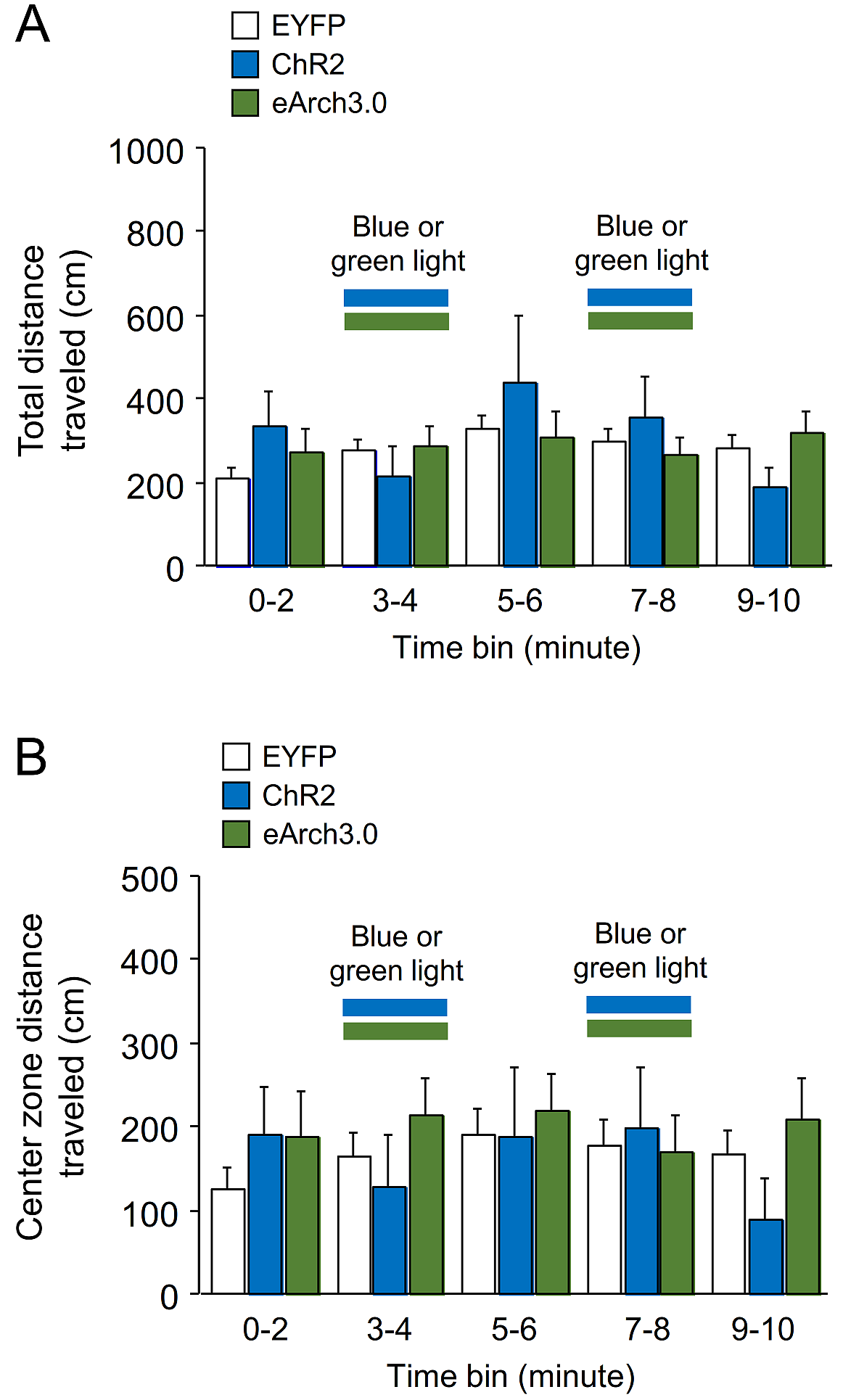

Supplement: Extended Data Figure 6-2 — In vivo photostimulation and photosilencing of transfected BA INs and PNs during a novel open field test. A, Neither photoexcitation in the ChR2 group nor photosilencing in the eArch3.0 group altered total distance traveled, relative to EYFP controls. B, Neither photoexcitation in the ChR2 group nor photosilencing in the eArch3.0 group altered center zone distance traveled, relative to EYFP controls; n = 6–17 per group. Download Figure 6-2, TIF file. [file sup_enu-eN-NWR-0220-19-s04.tif]
